# Long‐term risks and benefits of oral anticoagulation in atrial fibrillation patients with cancer: A report from the GLORIA‐AF registry

**DOI:** 10.1111/eci.14347

**Published:** 2024-11-13

**Authors:** Meng Li, Bi Huang, Steven Ho Man Lam, Hironori Ishiguchi, Yang Liu, Brian Olshansky, Menno V. Huisman, Tze‐Fan Chao, Gregory Y. H. Lip

**Affiliations:** ^1^ Liverpool Centre for Cardiovascular Science at University of Liverpool Liverpool John Moores University, and Liverpool Heart and Chest Hospital Liverpool UK; ^2^ Department of Cardiology, Dongzhimen Hospital Beijing University of Chinese Medicine Beijing China; ^3^ Department of Cardiology The First Affiliated Hospital of Chongqing Medical University Chongqing China; ^4^ Division of Cardiology, Department of Medicine and Clinical Science Yamaguchi University Graduate School of Medicine, Ube Yamaguchi Japan; ^5^ The Second Affiliated Hospital of Nanchang University Nanchang China; ^6^ Division of Cardiology, Department of Internal Medicine University of Iowa Hospitals and Clinics Iowa City Iowa USA; ^7^ Department of Thrombosis and Hemostasis Leiden University Medical Center Leiden The Netherlands; ^8^ Division of Cardiology, Department of Medicine Taipei Veterans General Hospital Taipei Taiwan; ^9^ Institute of Clinical Medicine and Cardiovascular Research Center National Yang Ming Chiao Tung University Taipei Taiwan; ^10^ Department of Clinical Medicine Aalborg University Aalborg Denmark

**Keywords:** atrial fibrillation, cancers, major bleeding, oral anticoagulants, thromboembolism

## Abstract

**Background:**

Anticoagulation therapy in patients with atrial fibrillation (AF) and concomitant cancer can be challenging due to the significantly increased risk of both embolism and bleeding. Moreover, the benefits and risks of vitamin K antagonists (VKA, eg. warfarin) versus non‐vitamin K antagonist oral anticoagulants (NOACs) in such patients are less well understood.

**Methods:**

From the prospective, global, multi‐centered Global Registry on Long‐Term Antithrombotic Treatment in Patients with Atrial Fibrillation (GLORIA‐AF), we characterized these patients according to their history of prior cancer when enrolled. All patients received anticoagulant therapy. The primary outcome was the composite of all‐cause mortality, stroke, transient ischemic attack, systemic embolism. The secondary endpoints were all‐cause mortality, cardiovascular death, stroke, major bleeding and thromboembolism during the 3 years follow‐up period. Cox regression analyses were used to calculate the hazard ratio (HR) and confidence interval (CI) following propensity score matching (PSM).

**Results:**

Overall, among 16,700 patients enrolled in Phase III in GLORIA‐AF, 1725 (10%) patients had concomitant cancer(s) at enrolment. After PSM, the primary outcome occurred in 250 (14.8%) of patients with cancer(s) and 160 (9.3%) without cancer(s) (HR, 1.62 [95% CI, 1.33–1.97], *p* < .001) during the 3 years follow‐up period. The risk of all‐cause mortality was significantly higher in patients with cancer(s) versus non‐ cancer(s) (HR, 1.71 [95% CI, 1.37–2.12], *p* < .001). In patients with cancer(s), after PSM, the use of NOACs was associated with reduced risk of the primary outcome compared with that of VKA (HR, .69 [95% CI, .49–.99], *p* = .043), as well as a lower risk of thromboembolism (HR, .49 [95% CI, .24–1.00], *p* = .051), but the risk of major bleeding was not significantly different (HR, .87 [95% CI, .48–1.56], *p* = .635). Subgroup analysis in patients with cancers showed a reduced risk of major bleeding with NOACs compared with VKA (HR, .18 [95% CI, .04–.8], *p* = .024) in patients with coronary artery disease (CAD). For the main cancer subtypes (genitourinary, breast, gastrointestinal, haematological and skin), the trends for the risk of primary outcome were consistently favouring NOACs compared with VKA without any significant interaction among these five cancers.

**Conclusions:**

Cancer is a common comorbidity in patients with AF and is associated with increased risk of composite of all‐cause mortality and thromboembolism. Compared with VKA, NOACs was associated with a lower risk of composite events and showed an advantage in lower risk of thromboembolism, as well as a reduced risk of major bleeding when CAD was also present.

## Graphical abstract

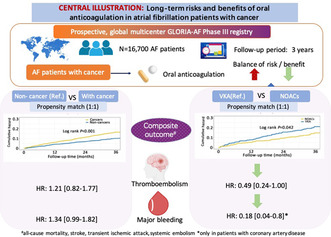

## 1 INTRODUCTION

Growing scientific evidence indicates a bidirectional link between AF and cancer.^1^, ^2^, ^3^ Approximately 14%–24% of AF patients had a concomitant diagnosis of cancer.^2^, ^3^ Moreover, in patients with cancer, the incidence of AF is as high as 30%–46% in some studies,^2^, ^4^, ^5^ largely contributed by the cancer itself, cancer treatment (chemotherapy and/or radiotherapy), and pre‐existent cardiovascular comorbidities.^6^, ^7^ Aging, common shared risk factors, and genetic background contribute to the cooccurrence of the two morbidities.^2^, ^6^

The coexistence of AF and cancer may lead to increased risk of major bleeding, thromboembolic events,^8^, ^9^, ^10^ and higher mortality compared to those cancer patients who did not develop AF.^11^ Therefore, management and treatment of AF patients with cancer remains a great challenge for clinicians. On one hand, cancer‐specific and therapy‐related factors may lead to an increased risk of arterial thromboembolism and stroke^12^; whilst on the other hand, physicians may hesitate to give appropriate treatment, including anticoagulant therapy to patients with AF and concomitant cancer for fear of increasing the bleeding risk although cancer is associated with a hypercoagulable state.^13^ This decision making is also complicated by patients with AF being clinically complex, with a high prevalence of frailty, multimorbidity and polypharmacy, all of which affects treatments and outcomes.^14^, ^15^, ^16^ Given the distinct risk–benefit profile of anticoagulation in cancer patients, the decision regarding selection of antithrombotic therapy for AF patients with cancer remains a challenge.^17^

To date, however, scant data are available on establishing the safety and efficacy of long‐term oral anticoagulation (OAC) therapy in prospective cohorts of AF patients with cancer. The aim of the current study was to assess long‐term risks and benefits of optimizing OAC among AF patients with cancer in a prospective, global multicenter registry, the Global Registry on Long‐Term Antithrombotic Treatment in Patients with Atrial Fibrillation (GLORIA‐AF) Phase III registry.

## 2 METHODS

### 2.1 Study design and population

GLORIA‐AF is a prospective, global registry of patients from 935 centers across 38 participating countries. Recently, a lot of articles related to the topic of this manuscript, including detailed designs and research results from different perspectives were published, which are summarized in Table S1. The present study focuses on patients form GLORIA‐AF phase III.^18^ In brief, adults (≥18 years) with a recent diagnosis of non‐valvular AF (i.e. within 3 or 4.5 months in Latin America) and a CHA_2_DS_2_‐VASc score ≥1 were consecutively enrolled. CHA2DS2‐VASc is still used for stroke risk stratification globally,^19^, ^20^ but considering current insights and updated guidelines, both CHA2DS2‐VASc and CHA2DS2‐VA score were analysed in this study. All patients received anticoagulant therapy. We characterized these patients according to their history of prior cancer when enrolled. The main exclusion criteria were the presence of mechanical heart valve (or patients expected to undergo valve replacement), prior treatment with vitamin K antagonist (VKA) for >60 days during lifetime, other clinical indication for OAC treatment, or life expectancy <1 year.^21^ The study protocol was approved by local institutional review boards at each participating center. The original studies were registered with ClinicalTrials.gov, NCT01468701.^21^

### 2.2 Study flowchart

**FIGURE 1:**
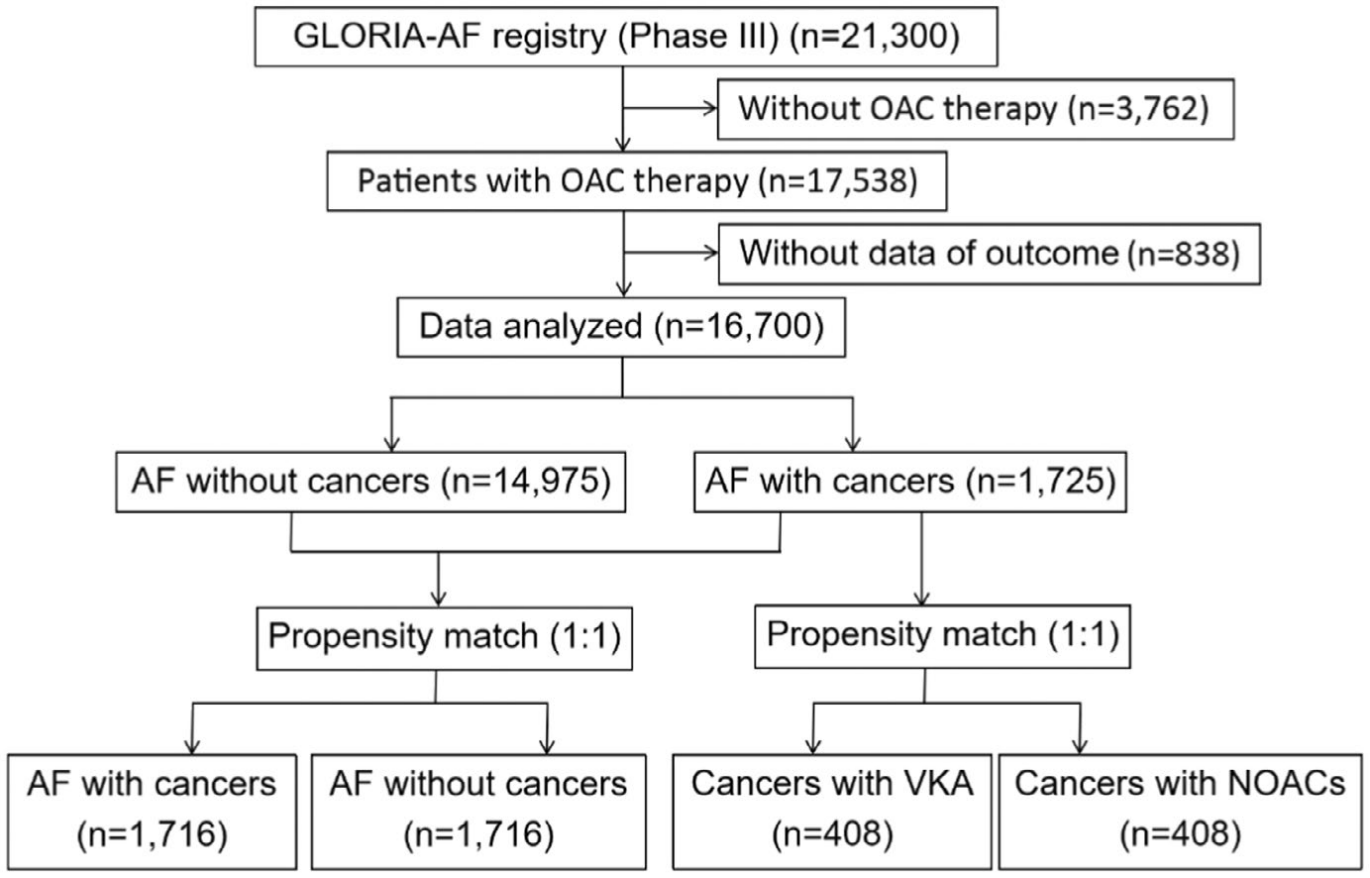
The study flowchart. AF, atrial fibrillation; NOACs, non‐vitamin K antagonist oral anticoagulants; VKA, vitamin K antagonist.

We first analysed the impact of cancers on the prognosis and then further evaluated the effectiveness and safety of VKA versus non‐vitamin K antagonist oral anticoagulants (NOACs) in AF patients with cancers with a propensity score matching (PSM) analysis (Figure 1).

### 2.3 Treatment, follow‐up and outcomes

During follow‐up, data on OAC discontinuation and major outcomes were recorded until study withdrawal, death, or the end of the study. We defined OAC nonpersistence as either discontinuation (switching to another antithrombotic regimen or a ≥ 30‐day interruption of the treatment received at baseline (to exclude temporary interruptions attributable to invasive procedures or surgery)) or study termination. Dose adjustments/reductions were not counted as discontinuation.

We defined our primary outcome as composite of all‐cause mortality, stroke, transient ischemic attack, systemic embolism. Secondary exploratory outcomes included: (i) all‐cause mortality, (ii) cardiovascular death, (iii) stroke, (iv) major bleeding (defined as a life‐threatening or fatal bleeding, symptomatic bleeding in a critical organ, or a bleeding associated with a haemoglobin reduction of ≥20 g/L or leading to ≥2 units of blood transfusion), and (v) thromboembolism (i.e. the composite of stroke, transient ischemic attack (TIA), and other non‐central nervous system (CNS) atrial embolism).^22^

### 2.4 Statistical analysis

Baseline characteristics were compared using independent‐sample t tests for continuous variables and *χ* ^2^ tests for categorical variables. To create balanced cohorts, we performed a PSM, using logistic regression. We performed a 1:1 greedy nearest neighbour matching model. Any baseline characteristic with an absolute standardized mean difference (SMD) between cohorts lower than .1 was considered well‐matched. We included the following variables in the PSM: age, sex, body mass index, comorbidities hypertension, coronary artery disease (CAD), chronic heart failure, diabetes, chronic kidney disease (CKD), left ventricular hypertrophy, hyperlipidaemia, peripheral artery disease, chronic obstructive pulmonary disease (COPD), previous stroke or TIA, history of bleeding, systolic blood pressure and heart rate at enrollment, embolism and bleeding scores and cardiovascular medications (including oral anticoagulants, antiplatelet, antiarrhythmics, digoxin, angiotensin converting enzyme inhibitor, angiotensin II receptor blocker, statins and diuretics).

Kaplan–Meier curves and log‐rank tests were used to compare the survival distributions during the follow‐up period. Cox proportional hazard regression was used to reveal the associations of cancer, and anticoagulation therapy with the adverse outcomes in the matched cohorts. Hazard ratios (HR) and 95% confidence intervals (CI) were calculated to quantify the association in A two‐sided *p* < .05 was considered statistically significant. All analyses were performed with R version 4.3.1 (R Core Team 2020, Vienna, Austria).

## 3 RESULTS

### 3.1 Incidence, baseline characteristics of cancer in patients with AF

**FIGURE 2:**
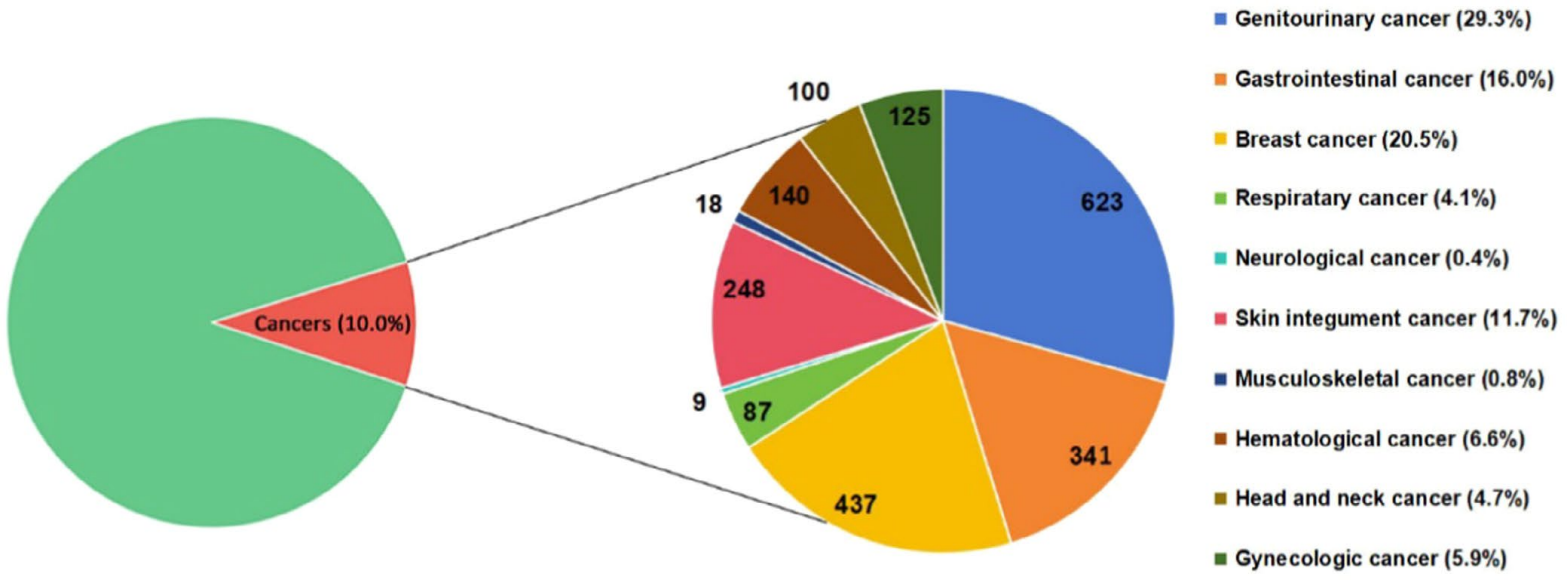
Distribution of cancers in GLORIA‐AF (Phase III).

Between January 2014 and December 2016, 21,300 patients were enrolled for GLORIA‐AF phase III, among which 2128 (10%) had concomitant cancer. A total of 10 types of cancer was reported, among which genitourinary cancer (29.3%), breast cancer (20.5%), and gastrointestinal cancer (16.0%) were the most common cancers (Figure 2).

In GLORIA‐AF phase III, 4600 patients were excluded due to lack of outcome data or non‐OAC use, and finally, the remaining 16,700 with OAC treatment patient population were analysed in this study, of whom 1725 (10.3%) had prior cancer (75 (69, 80) years; 56% males) and 14,975 were without cancer (‘non‐cancer’ group) (71 (64, 78) years; 55% males; Table 1).

**TABLE 1:** Baseline characteristics in AF patients with and without cancers before and after propensity matching.

	Before propensity score match			After propensity score match		
	AF without cancers (*n* = 14,975)	AF with cancers (*n* = 1725)	SMD	AF without cancers (*n* = 1716)	AF with cancers (*n* = 1716)	SMD
Age (years)	71 (64, 78)	75 (69, 80)	.490	75 (69, 80)	75 (69, 80)	.017
Male (%)	8250 (55%)	959 (56%)	.010	966 (56%)	965 (56%)	.002
BMI (kg/m^2^)	27.8 (24.8, 31.9)	27.6 (24.3, 31.6)	.081	27.6 (24.7, 31.3)	27.5 (24.3, 31.5)	.008
Comorbidities (*n*, %)						
Hypertension	11,431 (76%)	1318 (76%)	.002	1312 (76%)	1322 (76%)	.038
Coronary artery disease	2568 (17%)	366 (21%)	.100	352 (20%)	367 (21%)	.020
Chronic heart failure	3287 (22%)	335 (19%)	.064	324 (19%)	333 (19%)	.009
Diabetes	3601 (24%)	395 (23%)	.027	416 (24%)	394 (23%)	.018
Chronic kidney disease	366 (2.4%)	62 (3.6%)	.062	51 (2.9%)	66 (3.8%)	.013
Left ventricular hypertrophy	2876 (19%)	304 (18%)	.042	317 (18%)	306 (18%)	.034
Hyperlipidaemia	6164 (41%)	815 (47%)	.122	799 (46%)	815 (47%)	.006
Peripheral artery disease	430 (2.9%)	68 (3.9%)	.055	67 (3.9%)	68 (3.9%)	.018
COPD	874 (5.8%)	149 (8.6%)	.100	144 (8.3%)	147 (8.5%)	.002
Previous stroke/TIA	2084 (14%)	267 (15%)	.043	291 (17%)	268 (15%)	.002
History of bleeding	690 (4.6%)	143 (8.3%)	.134	132 (7.6%)	141 (8.1%)	.036
Vital signs at enrollment						
Systolic blood pressure (mmHg)	130 (120, 142)	130 (120, 142)	.020	130 (120, 142)	130 (120, 142)	.024
Heart rate (beats/minute)	76 (65, 90)	75 (64, 89)	.035	76 (64, 88)	75 (64, 89)	.028
Type of AF			.090			.016
Paroxysmal AF	7927 (53%)	992 (58%)		990 (57%)	997 (57%)	
Persistent AF	5471 (37%)	575 (33%)		584 (34%)	577 (33%)	
Permanent AF	1577 (11%)	158 (9.2%)		161 (9.3%)	161 (9.3%)	
Embolism and bleeding scores (*n*, %)						
CHA2DS2‐VASc> = 2	13,030 (87%)	1600 (93%)	.222	1615 (93%)	1608 (93%)	.016
CHA2DS2‐VA > =2	14,710 (98%)	1709 (99%)	.079	1707 (99%)	1704 (99%)	.022
HAS‐BLED> = 3	1942 (13%)	301 (17%)	.118	319 (18%)	310 (18%)	.003
Medications (*n*, %)						
Oral anticoagulants						
VKA	4131 (28%)	459 (27%)	.022	449 (26%)	458 (26%)	.015
Dabigatran	3433 (23%)	267 (15%)	.206	278 (16%)	269 (16%)	.016
Rivaroxaban	3439 (23%)	393 (23%)	.004	399 (23%)	396 (23%)	.046
Apixaban	3710 (25%)	575 (33%)	.182	574 (33%)	578 (33%)	.012
Edoxaban	262 (1.7%)	31 (1.8%)	.004	35 (2.0%)	34 (2.0%)	.009
Antiplatelet	2308 (15%)	353 (20%)	.125	311 (18%)	337 (20%)	.039
Digoxin	1232 (8.2%)	125 (7.2%)	.038	137 (7.9%)	126 (7.3%)	.034
ACEI	10,404 (68%)	1192 (68%)	.003	1222 (70%)	1185 (68%)	.003
ARB	11,198 (74%)	1347 (77%)	.080	1341 (77%)	1337 (77%)	.047
Statins	6810 (45%)	816 (47%)	.037	772 (44%)	807 (47%)	.020
Diuretics	6029 (40%)	664 (38%)	.031	624 (36%)	657 (38%)	.012

Abbreviations: ACEI, angiotensin converting enzyme inhibitor; AF, atrial fibrillation; ARB, angiotensin receptor blocker; BMI, body mass index; COPD, chronic obstructive pulmonary disease; SMD, standardized mean difference; TIA, transient ischemic attack; VKA, vitamin K antagonist.

Before PSM, AF patients with cancer had a higher prevalence of CAD, CKD, COPD, history of bleeding, embolism and bleeding scores (Table 1). After PSM, 1716 patients were identified for each group and the SMDs for all the variables between each group are less than .1 (Table 1). The baseline covariates were well‐balanced between patients with and without cancer.

### 3.2 Risk of follow‐up events on OAC in AF patients with cancer and non‐cancer

After PSM, during the follow‐up period of 3 years, the incidence of primary outcome in patients with and non‐cancer were 14.8% (250/1716) and 9.3% (160/1716), respectively.

**FIGURE 3:**
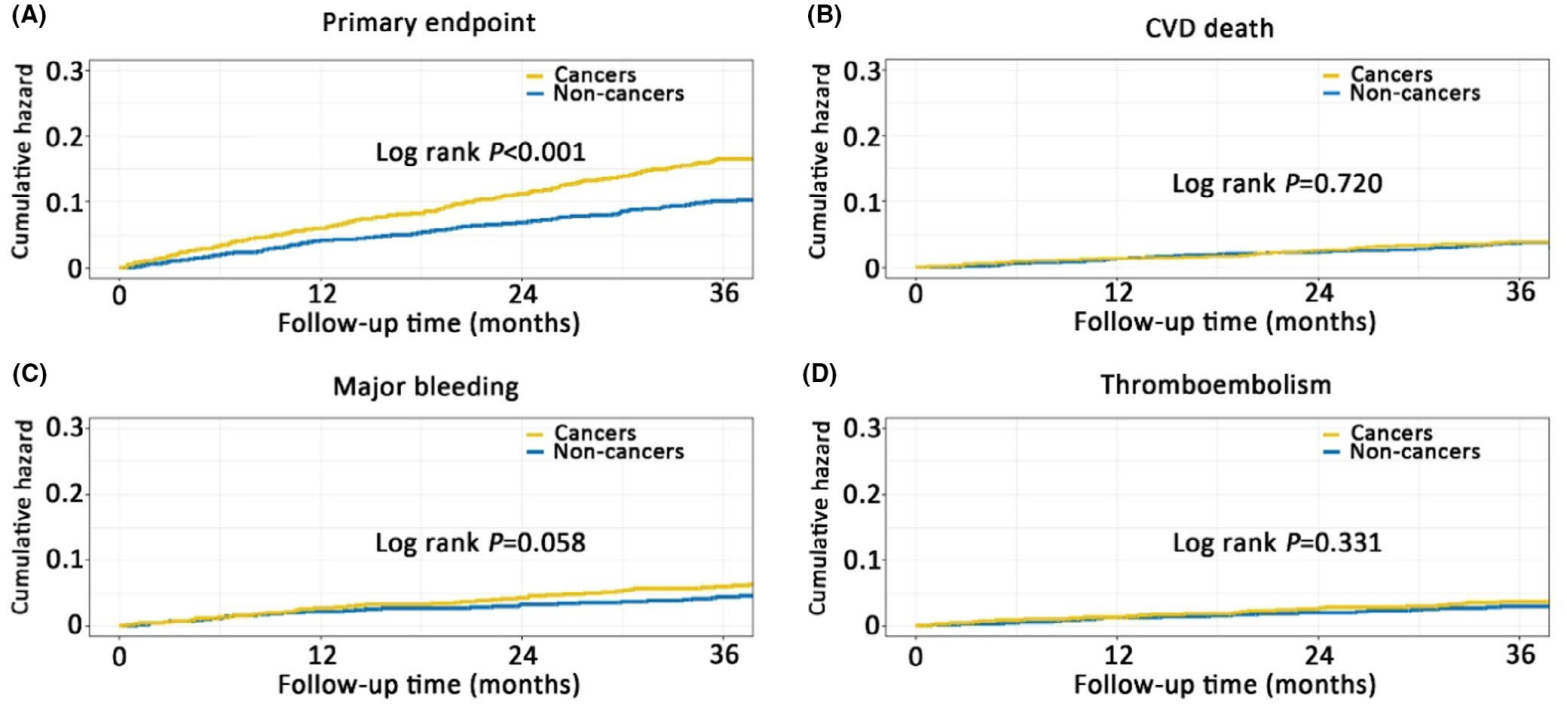
Cumulative event curve in cancer and non‐cancers. (A) Cumulative event curve for primary endpoint in cancer and non‐cancers; (B) Cumulative event curve for CVD death in cancer and non‐cancers; (C) Cumulative event curve for major bleeding in cancer and non‐cancers; (D) Cumulative event curve for thromboembolism in cancer and non‐cancers. CVD, cardiovascular disease.

AF patients with cancers were associated with significant increased risks of primary endpoint (HR, 1.62 [95% CI, 1.33–1.97], *p* < .001) and all‐cause mortality (HR, 1.71 [95% CI, 1.37–2.12], *p* < .001) compared with those without cancers. The risks of cardiovascular death (HR, 1.07 [95% CI, .75–1.53], *p* = .717), stroke (HR, 1.18 [95% CI, .76–1.82], *p* = .463), major bleeding (HR, 1.34 [95% CI, .99–1.82], *p* = .059) and thromboembolism (HR, 1.21 [95% CI, .82–1.77], *p* = .333) were not statistically significant between cancer patients and non‐cancer patients (Table 2 and Figure 3).

**TABLE 2:** Endpoints in patients with and without cancers.

Outcomes	No. of events (%)	No./100 pts/year (95% CI)	HR (95% CI)	*p‐*value
Primary endpoint				
Patients without cancers (*n* = 1716)	160 (9.3%)	3.28 (2.80–3.83)		
Patients with cancers (*n* = 1716)	250 (14.8%)	5.31 (4.68–6.01)	1.62 (1.33–1.97)	<.001
Secondary endpoints				
All‐cause mortality				
Patients without cancers (*n* = 1716)	127 (7.4%)	2.57 (2.15–3.06)		
Patients with cancers (*n* = 1716)	212 (12.4%)	4.38 (3.81–5.01)	1.71 (1.37–2.12)	<.001
Cardiovascular death				
Patients without cancers (*n* = 1716)	58 (3.4%)	1.17 (.89–1.51)		
Patients with cancers (*n* = 1716)	61 (3.6%)	1.24 (.95–1.60)	1.07 (.75–1.53)	.717
Stroke				
Patients without cancers (*n* = 1716)	38 (2.2%)	.77 (.54–1.05)		
Patients with cancers (*n* = 1716)	44 (2.6%)	.90 (.66–1.21)	1.18 (.76–1.82)	.463
Major bleeding				
Patients without cancers (*n* = 1716)	72 (4.2%)	1.47 (1.15–1.86)		
Patients with cancers (*n* = 1716)	95 (5.5%)	1.98 (1.60–2.42)	1.34 (.99–1.82)	.059
Thromboembolism (any stroke, TIA, or non‐CNS atrial embolism)				
Patients without cancers (*n* = 1716)	48 (2.8%)	.98 (.72–1.29)		
Patients with cancers (*n* = 1716)	57 (3.3%)	1.18 (.89–1.52)	1.21 (.82–1.77)	.333

Abbreviations: CI, confidence interval; CNS, central nervous system; HR, hazard ratio; TIA, transient ischemic attack.

### 3.3 Subgroup analysis in risk of outcomes in AF patients with cancer

In exploratory analysis of the endpoints, patients were divided into 14 subgroups stratified by cancer. This shows that the composite of primary outcome and thromboembolism were consistent in different subgroups with no significant interactions among different subgroups, but for major bleeding, there was interaction between cancers and CKD (CKD, HR .36 [95% CI, .10–1.33], non‐CKD, 1.48 [95% CI, 1.08–2.04], *p*_−interaction_ = .038) (Figures S1–S3).

### 3.4 Impact of NOACs versus VKA in AF patients with cancer

We analysed the effectiveness and safety of VKA users versus NOACs users in cancer patients. Table 3 shows baseline characteristics in cancer patients treated with VKA and NOACs. Before PSM, there were 459 patients (75 (70, 80) years; 59% males) treated with VKA and 1266 (75 (69, 80) years; 55% males) treated with NOACs. After PSM, there were 408 patients in both treatment groups and the baseline characteristics in both groups were closely balanced (Table 4).

**TABLE 3:** Baseline characteristics in cancer patients treated with VKA and NOACs before and after propensity matching.

	Before propensity score match			After propensity score match		
	VKA (*n* = 459)	NOACs (*n* = 1266)	SMD	VKA (*n* = 408)	NOACs (*n* = 408)	SMD
Age (years)	75 (70, 80)	75 (69, 80)	.082	75 (69, 80)	75 (70, 80)	.064
Male (%)	269 (59%)	690 (55%)	.082	232 (57%)	233 (57%)	.005
BMI (kg/m^2^)	27.5 (24.4, 31.3)	27.6 (24.3, 31.6)	.035	27.6 (24.8, 31.6)	27.6 (24.5, 31.1)	.031
Comorbidities (*n*, %)						
Hypertension	354 (77%)	964 (76%)	.023	312 (76%)	323 (79%)	.063
Coronary artery disease	110 (24%)	256 (20%)	.093	94 (23%)	89 (22%)	.031
Chronic heart failure	109 (24%)	226 (18%)	.154	86 (21%)	83 (20%)	.019
Diabetes	111 (24%)	284 (22%)	.042	100 (25%)	106 (26%)	.035
Chronic kidney disease	36 (7.8%)	26 (2.1%)	.408	13 (3.2%)	13 (3.2%)	.000
Left ventricular hypertrophy	101 (22%)	203 (16%)	.163	83 (20%)	83 (20%)	.000
Hyperlipidaemia	212 (46%)	603 (48%)	.029	189 (46%)	198 (49%)	.044
Peripheral artery disease	25 (5.4%)	43 (3.4%)	.113	18 (4.4%)	20 (4.9%)	.027
COPD	40 (8.7%)	109 (8.6%)	.004	36 (8.8%)	40 (9.8%)	.035
Previous stroke/TIA	63 (14%)	204 (16%)	.065	61 (15%)	68 (17%)	.047
History of bleeding	35 (7.6%)	108 (8.5%)	.032	28 (6.9%)	40 (9.8%)	.105
Vital signs at enrollment						
Systolic blood pressure (mmHg)	130 (120, 142)	130 (120, 142)	.048	130 (120, 142)	130 (120, 142)	.015
Heart rate (beats/min)	78 (66, 90)	74 (64, 88)	.100	77 (65, 90)	77 (65, 90)	.052
Type of AF			.282			.015
Paroxysmal AF	217 (47%)	775 (61%)		207 (51%)	217 (53%)	
Persistent AF	186 (41%)	389 (31%)		162 (40%)	138 (34%)	
Permanent AF	56 (12%)	102 (8.1%)		39 (9.6%)	53 (13%)	
Embolism and bleeding scores (*n*, %)						
CHA2DS2‐VASc> = 2	434 (95%)	1166 (92%)	.091	383 (94%)	387 (95%)	.036
CHA2DS2‐VA > =2	452 (98%)	1242 (98%)	.084	406 (99%)	405 (99%)	.031
HAS‐BLED> = 3	86 (19%)	215 (17%)	.047	67 (16%)	70 (17%)	.020
Medications (*n*, %)						
Antiplatelet	94 (20%)	293 (23%)	.073	87 (21%)	81 (20%)	.036
Digoxin	43 (9.4%)	82 (6.5%)	.118	35 (8.6%)	29 (7.1%)	.060
ACEI	288 (63%)	886 (70%)	.158	266 (65%)	256 (63%)	.054
ARB	358 (78%)	973 (77%)	.027	312 (76%)	315 (77%)	.017
Statins	213 (46%)	593 (47%)	.009	189 (46%)	199 (49%)	.049
Diuretics	193 (42%)	466 (37%)	.109	165 (40%)	170 (42%)	.025

Abbreviations: ACEI, angiotensin converting enzyme inhibitor; AF, atrial fibrillation; ARB, angiotensin receptor blocker; BMI, body mass index; COPD, chronic obstructive pulmonary disease; NOACs, non‐vitamin K antagonist oral anticoagulants; SMD, standardized mean difference; TIA, transient ischemic attack; VKA, vitamin K antagonist.

**TABLE 4:** Endpoints in cancer patients treated with VKA and NOACs.

Outcomes	No. of events (%)	No./100 pts/year (95% CI)	HR (95% CI)	*p*‐value
Primary endpoint				
Patients with VKA (*n* = 408)	74 (18.1%)	6.66 (5.23–8.36)		
Patients with NOACs (*n* = 408)	53 (13.0%)	4.66 (3.49–6.09)	.69 (.49–.99)	.043
Secondary endpoints				
All‐cause mortality				
Patients with VKA (*n* = 408)	58 (14.2%)	5.07 (3.85–6.55)		
Patients with NOACs (*n* = 408)	46 (11.3%)	4.01 (2.93–5.35)	.78 (.53–1.15)	.215
Cardiovascular death				
Patients with VKA (*n* = 408)	12 (2.9%)	1.03 (.53–1.80)		
Patients with NOACs (*n* = 408)	15 (3.7%)	1.29 (.72–2.13)	1.24 (.58–2.64)	.582
**S**troke				
Patients with VKA (*n* = 408)	13 (3.2%)	1.12 (.60–1.92)		
Patients with NOACs (*n* = 408)	5 (1.2%)	.43 (.14–1.00)	.38 (.13–1.06)	.064
Major bleeding				
Patients with VKA (*n* = 408)	24 (5.9%)	2.11 (1.35–3.15)		
Patients with NOACs (*n* = 408)	21 (5.1%)	1.85 (1.15–2.83)	.87 (.48–1.56)	.635
Thromboembolism (any stroke, TIA, or non‐CNS atrial embolism)				
Patients with VKA (*n* = 408)	22 (5.4%)	1.93 (1.21–2.93)		
Patients with NOACs (*n* = 408)	11 (2.7%)	.95 (.48–1.70)	.49 (.24–1.00)	.051

Abbreviations: CI, confidence interval; CNS, central nervous system; HR, hazard ratio; NOACs, non‐vitamin K antagonist oral anticoagulants; TIA, transient ischemic attack; VKA, vitamin K antagonist.

In patients with cancer, after PSM, the incidence of primary outcome in patients with NOACs and VKA were 13.0% (53/408) and 18.1% (74/408), respectively. NOACs was associated with reduced risk of primary outcome compared with VKA (HR, .69 [95% CI, .49–.99, *p* = .043]).

**FIGURE 4:**
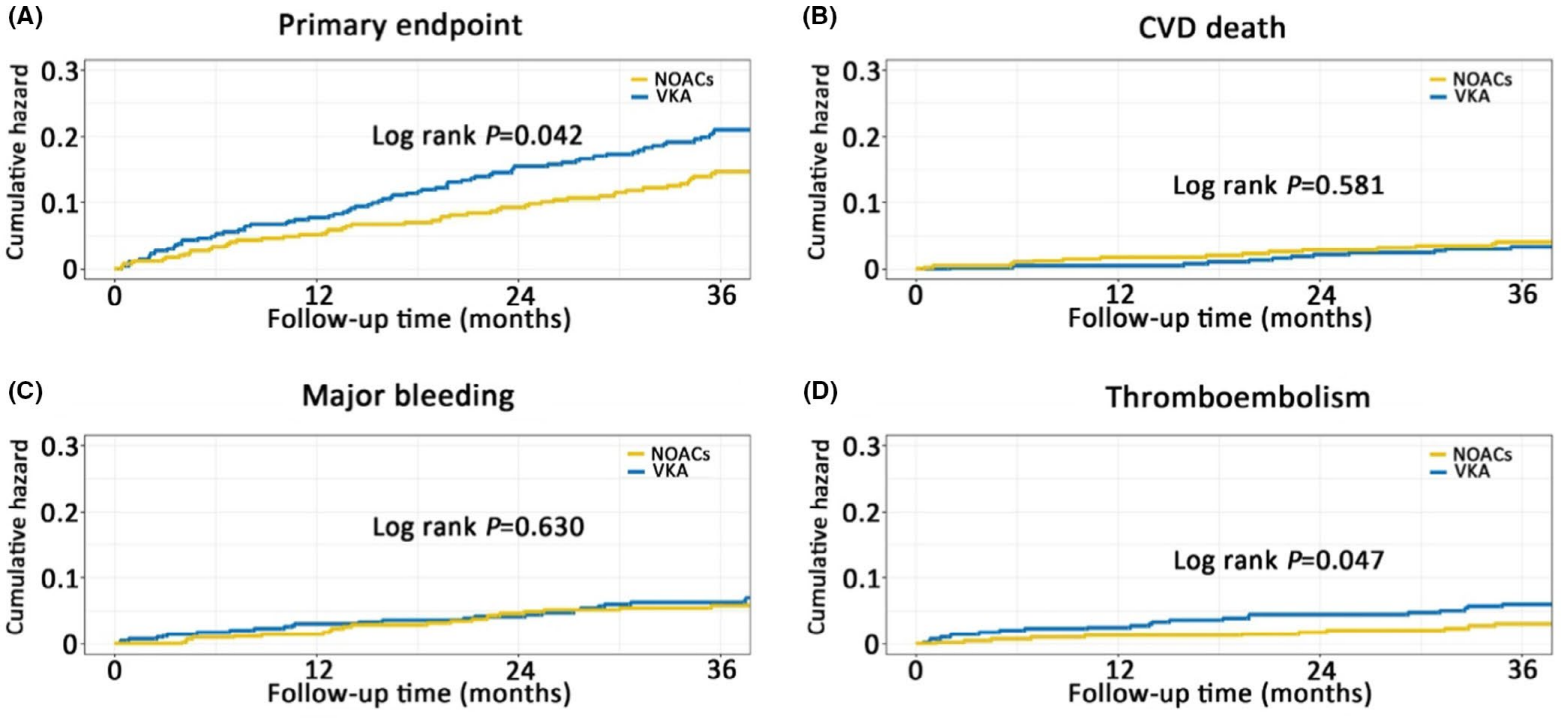
Cumulative event curve in patients treated with VKA and NOACs. (A) Cumulative event curve for primary endpoint in patients treated with VKA and NOACs; (B) Cumulative event curve for CVD death in patients treated with VKA and NOACs; (C) Cumulative event curve for major bleeding in patients treated with VKA and NOACs; (D) Cumulative event curve for thromboembolism in patients treated with VKA and NOACs. CVD, cardiovascular disease; NOACs, non‐vitamin K antagonist oral anticoagulants; VKA, vitamin K antagonist.

NOACs was associated with a reduced long‐term thromboembolism risk compared with VKA (HR, .49 [95% CI, .24–1.00], *p* = .051), but the risk of all‐cause mortality (HR, .78 [95% CI, .53–1.15], *p* = .215), cardiovascular death (HR, 1.24 [95% CI, .58–2.64], *p* = .582), stroke (HR, .38 [95% CI, .13–1.06], *p* = .064), and major bleeding (HR, .87 [95% CI, .48–1.56], *p* = .635) were not significantly different (Table 4 and Figure 4).

### 3.5 Subgroup analysis of NOACs versus VKA in AF patients with cancer

In the exploratory analysis the association between anticoagulants and the various endpoints were fairly consistent in different subgroups except that in patients with CAD, where NOACs was associated with reduced risk of major bleeding compared with VKA (HR for CAD, .18 [95% CI, .04–.8] while the HR for non‐CAD was 1.48 [95% CI, .73–2.99], respectively, *p*_−interaction_ = .012) (Figures S4–S6).

### 3.6 Subgroup analysis of NOACs versus VKA in main cancer subtypes

Due to the relatively small sample size, its statistical power is low, therefore, this study just conducted subgroup of the five most common cancers (genitourinary, breast, gastrointestinal, haematological and skin). A pooled analysis for the risk of thromboembolism in main cancer types found that the risk of thromboembolism in gastrointestinal cancer is lower compared to non‐gastrointestinal cancer (HR, .35 [95% CI, .13–.99], *p* = .047) and no statistically significant was found in other cancers (Table 5).

**TABLE 5:** Risk of thromboembolism (any stroke, TIA or non‐CNS atrial embolism) in different cancers.

Cancers	HR	95% CI	*p*‐value
Breast cancer versus non‐breast cancer	1.31	.57–3.01	.581
Gastrointestinal cancer versus non‐gastrointestinal cancer	.35	.13–.99	.047
Genitourinary cancer versus non‐genitourinary cancer	1.22	.64–2.33	.620
Skin integument cancer versus non‐skin integument cancer	2.83	.36–21.9	.351
Haematological cancer versus non‐haematological cancer	.59	.14–2.51	.552

**FIGURE 5:**
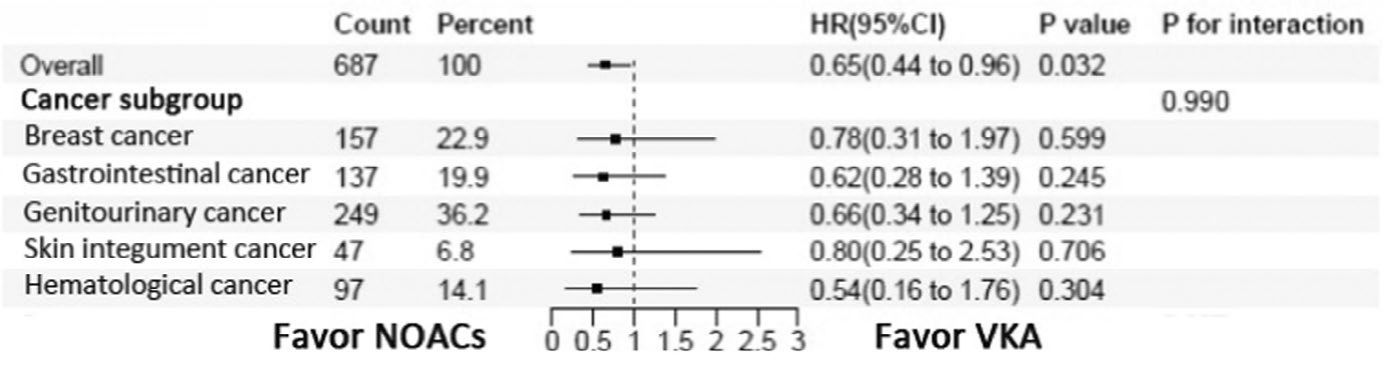
Subgroup analysis of the primary endpoint in cancer patients treated with VKA and NOACs. NOACs, non‐vitamin K oral anticoagulants; VKA, vitamin K antagonist.

The chances of having the primary outcome consistently favoured NOACs over VKA without any significant interactions among these five cancers (Figure 5).

## 4 DISCUSSION

In this study our main findings are as follows: (i) cancer was reported in 1 out of 10 patients with non‐valvular AF; (ii) cancer was independently associated with increased risk of the composite outcome and all‐cause mortality during long‐term OAC therapy; (iii) compared with VKA, the use of NOACs was associated with a reduced risk of the primary composite outcome and showed a lower risk of thromboembolism, as well as a reduced risk of major bleeding in the subgroup patients with CAD.

Previous studies have highlighted the close interrelation between AF and cancer.^2^, ^23^, ^24^ Analyses from Austria and the United States respectively showed 13.7%^2^ and 23.8%^3^ of patients with AF had a concomitant diagnosis of cancer. The prevalence of cancer is 10% in our present prospective global cohort of AF patients, which was slightly lower than the two studies from Austria and the Unites States.^2^, ^3^ Our global prevalence of cancer may differ from nationwide prevalence due to regional variation, ethnic disparities and other factors, such as cancer types, cancer status (active or not) and age groups.^2^, ^25^

Cancer shares several common risk factors with AF, such as elderly, smoking, alcohol abuse, and obesity.^26^, ^27^, ^28^ Additionally, some mechanisms are suggested as possible causes or triggers for the pathogenesis of both conditions, including a systemic proinflammatory state in patients with cancer that may lead to atrial remodelling,^29^ cardiovascular toxicity of numerous cancer therapy (chemotherapy and/or radiotherapy),^6^ and a high perioperative risk of AF in patients undergone cancer surgery,^30^ which usually lead to coexistence of cancer and AF, making management options more complex.

Previous studies in AF patients have shown that cancer was associated with an increased risk of all‐cause mortality.^31^, ^32^ Two studies conducted in France found that cancer increased all‐cause mortality (HR, 2.00 [95%CI, 1.99–2.01])^31^ and was even the strongest predictor of all‐cause mortality (OR, 1.81 [95%CI, 1.78–1.85]) in patients with AF.^32^ Our study shows that AF patients with cancer faced an increased risk of the composite of all‐cause mortality and thromboembolism, but not an increased in thromboembolism risk. The different types of cancer have their respective natural course and prognosis; although they coexist with AF, the outcome is mainly dependent on the cancer itself especially in those with low grade cancers.^33^

Besides, studies have shown that patients with cancers had significant increased risk of both bleeding and thromboembolism.^31^, ^34^ The balance between thromboembolic and bleeding risks is particularly a challenge to decision‐making.^35^ Cancer induces a prothrombotic state^36^ and may further increase the risk of thrombotic events in patients with AF.^3^ Meanwhile, some malignancies and cancer therapies may increase the bleeding risk among AF patients with anticoagulant therapy.^37^ In subgroups analysis in patients with CKD, there was interaction between cancers and CKD and HR for higher major bleeding in only non‐CKD was statistically significant. The majority of oral anticoagulants (including NOACs) are partly renally cleared,^38^ CKD patients also have a higher risk of bleeding.^38^, ^39^ In the present study, only 122 people were comorbid with CKD, accounting for only 3.6% of the whole group. The small sample size, which limits its statistical power, calls for further research to study this important group.

However, in this study, we found that during the 3 years follow‐up, patients with cancer on OAC therapy had nonsignificant increased risk of major bleeding. According to current ESC cardio‐oncology Guidelines, patients with AF and cancer and a CHA_2_DS_2_‐VASc score of 0 (men) or 1 (women) may be considered for anticoagulant therapy after assessment of the bleeding risk (HAS‐BLED score recommended).^35^ The effectiveness and safety of NOACs have been shown to be non‐inferior or even superior to VKA in patients with non‐valvular AF and concomitant cancer.^40^, ^41^, ^42^, ^43^ In our current study, compared with VKA, the use of NOACs was associated with a reduced risk of composite events, largely due to reducing the burden of systemic embolism.^44^

With regard to the risk of long‐term major bleeding, from this study, NOACs was comparable compared with VKA. Though, our subgroup analysis showed AF patients with CAD, compared with VKA, NOACs was associated with a reduced risk of major bleeding. Similar findings were found in two meta‐analysis (albeit not specifically in cancer populations) which showed that dual‐antithrombotic therapy with NOACs was associated with lower risk of major bleeding^45^, ^46^ compared with triple‐antithrombotic therapy with VKA, but not with dual‐antithrombotic therapy with VKA.^47^

However, the comparisons of NOACs and VKA regarding risk of major bleeding in patients with AF with cancer remain inconsistent and varied among NOAC‐NOAC comparisons. For example, a nationwide cohort study^48^ found that NOACs (apixaban, dabigatran, edoxaban and rivaroxaban) were associated with a significant reduction of major bleeding risks when compared with warfarin in patients with AF with cancer. In the ARISTOPHANES study,^49^ when compared with warfarin, apixaban was associated with a lower risk of major bleeding; while dabigatran and rivaroxaban had similar risks of major bleeding. Currently, NOACs are recommended by current guidelines in preference to VKA in patients without a high bleeding risk, significant drug–drug interactions or severe renal dysfunction.^35^ Additionally, the HAS‐BLED score, seems to underestimate the bleeding risk in patients with AF and cancer^31^ and a HAS‐BLED score of ≥3 was a poor discriminator for high bleeding risk in AF and cancer patients.^50^

Current data indicate that the risks of thromboembolism and bleeding in patients with AF and cancer differ among different types of cancer.^51^ A systematic review indicated that cancer mainly increased the risk of bleeding among AF patients, especially with breast cancer.^52^ In other studies, patients with hematologic and lung cancer were associated with an increased risk of major bleeding irrespective of the CHA2DS2‐VASc category and use of anticoagulation,^53^ while respiratory tract cancer had a higher tendency for thromboembolism.^54^ However, our study indicated that the risk of thromboembolism in gastrointestinal cancer is lower compared to non‐gastrointestinal cancer, duo to NOACs were adequately absorbed in cancer patients even after partial or total gastrectomy.^55^ Intermittent assessment of trough and peak levels should be considered to assure adequate absorption. Notably, the current guidelines have no recommendations for specific anticoagulation regimens in patients with cancer subtypes, given limited data in the field.^56^ Other studies had reported that AF patients with cancer (breast cancer, prostate cancer, or lung cancer) treated with NOACs experienced similar rates of stroke and major bleeding as those with VKAs,^57^, ^58^ while NOACs had lower risk of gastrointestinal bleeding and non‐critical site bleeding than VKAs.^58^ In our analysis, the trends for reduced risk of composite outcome consistently favoured NOACs over VKA without any significant interaction among the five most common cancers. Further studies are needed to clarify the optimal anticoagulants in this particular population.^35^

### 4.1 Strengths and limitations

Our study has analysed cancer comorbidity patterns on a large, contemporary and global real‐world prospective cohort of AF patients. Our present study indicates that the management of AF patients with concomitant cancers should focus on the risk of cancer itself, and taking standard OAC therapy may have the potential benefit for thromboembolism.

Nonetheless, we acknowledge some limitations. First, this study did not collect cancer‐related information prospectively. Therefore, we lacked data for the exact time of the diagnosis of cancer, cancer staging and grading, the tumour status (active or historical), whether patients developed new cancer during AF treatment and follow‐up. Second, the detailed treatment strategy and biomarkers were unavailable in this research. These could have impacted our results, as bleeding risks may change during the course of therapy and certain chemotherapy drugs used to treat cancer may interact with anticoagulants.^59^ Third, we could not conduct subgroup analysis by different NOACs subgroup and major bleeding and thromboembolism analysis of cancer subtype due to limited sample size. Also, the risks and benefits of different NOACs and cancer subtype may be varied.^49^ Lastly, the present trial included cancer patients aged 75 years or younger, whereas data concerning the ‘old’ group (patients aged >75 years) and the ‘oldest‐old’ group (patients aged >85 years) were scanty. Nevertheless, it is not uncommon to encounter in clinical practice cancer patients with AF, who are aged over 75 years old. The prevalence of both AF and cancer was 15%–20% in the ‘oldest‐old’ group in this study, and in prior papers, NOACs/VKAs have been prescribed in approximately 30% of such patients.^60^ Further studies should be conducted for evaluating both the efficacy and safety of NOACs versus VKAs in cancer patients aged over 75 years (in both the ‘old’ and ‘oldest‐old’ groups).

## 5 CONCLUSION

Cancer is a common comorbidity in patients with AF and is associated with increased risk of composite of all‐cause mortality and thromboembolism. Compared with VKA, NOACs was associated with a lower risk of composite events and showed a lower risk of thromboembolism, as well as a reduced risk of major bleeding when CAD was also present.

## AUTHOR CONTRIBUTIONS

Meng Li and Bi Huang conceived the study design and drafted the manuscript. Steven Ho Man Lam, Hironori Ishiguchi and Yang Liu analysed data. Brian Olshansky, Menno V Huisman and Tze‐Fan Chao reviewed and edited the manuscript. Gregory Y. H. Lip reviewed and edited the manuscript, and contributed to the study design. All authors read and approved the final manuscript.

## CONFLICT OF INTEREST STATEMENT

The authors declare no competing interests.

## CONSENT TO PARTICIPATE

All patients provided written informed consent.

## CONSENT FOR PUBLICATION

Not applicable.

## Supporting information


Figure S1:

Table S1:

## Data Availability

Data supporting this study were contributed by Boehringer Ingelheim and were made available through Vivli, Inc. Access was provided after a proposal was approved by an independent review committee identified for this purpose and after receipt of a signed data sharing agreement.
