# Supplementary material for: Long‐term risks and benefits of oral anticoagulation in atrial fibrillation patients with cancer: A report from the GLORIA‐AF registry
Source: Eur J Clin Invest. 2024 Nov 13;55(2):e14347. doi: 10.1111/eci.14347 (PMC11744914; doi:10.1111/eci.14347)
Supplement: Supplementary file 1 — Figure S1: [file ECI-55-e14347-s002.docx]

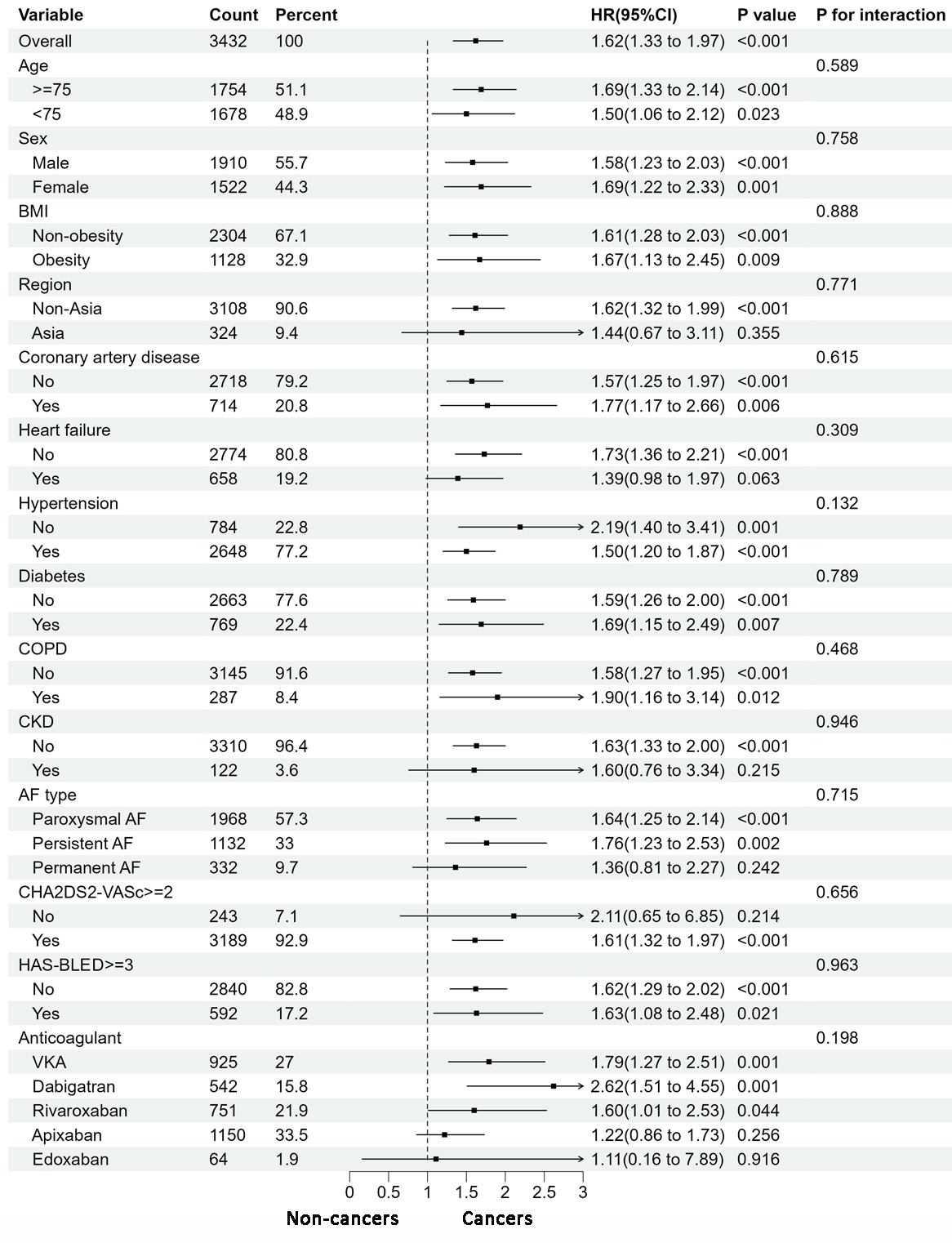


Supplement Figure 1: Subgroup analysis of the primary endpoint in patients with and without cancers.

AF, atrial fibrillation; BMI, body mass index; CI, confidence interval; COPD, chronic obstructive pulmonary disease; CKD, chronic kidney disease; HR, hazard ratio; VKA, vitamin K antagonist.


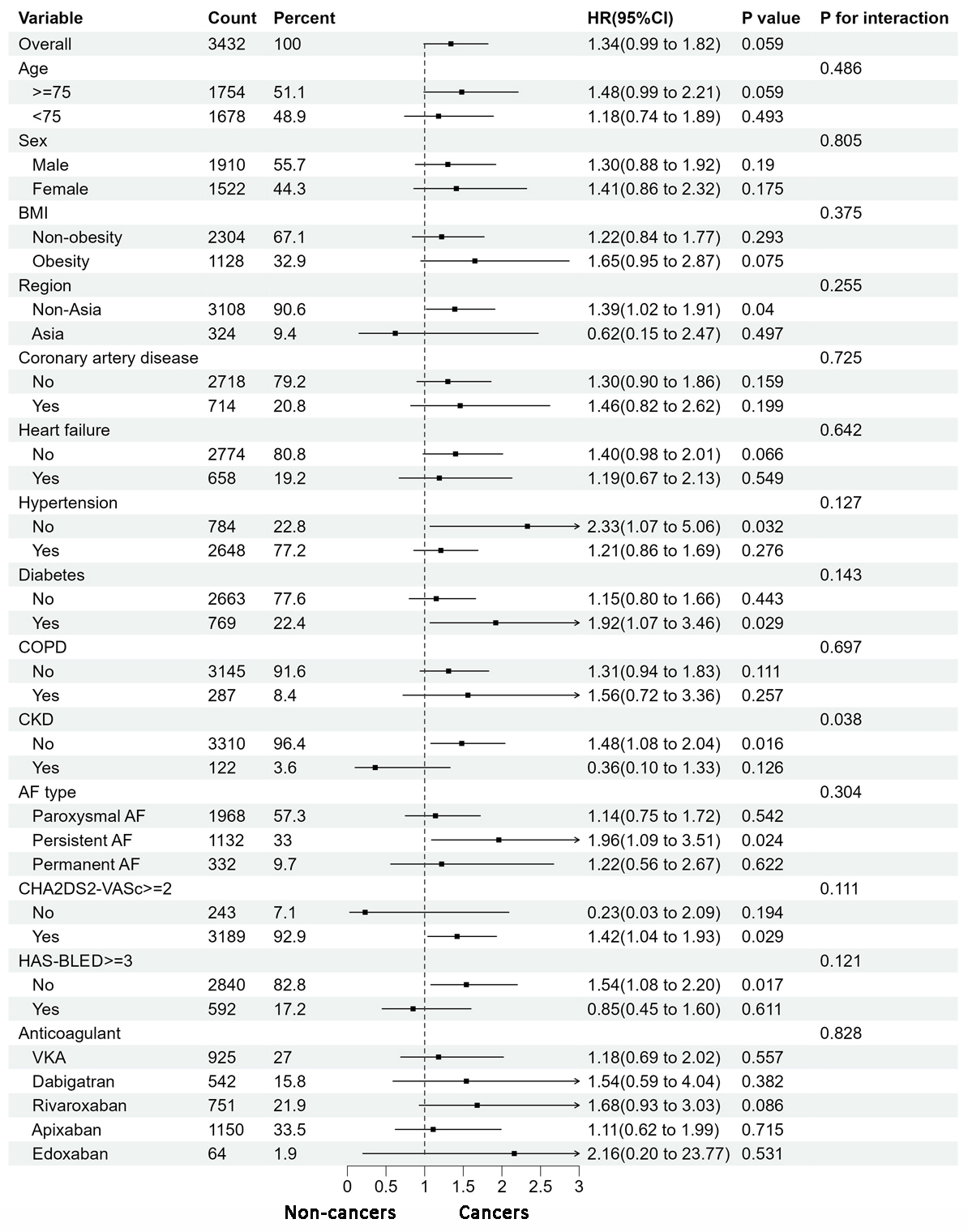


Supplement Figure 2: Subgroup analysis of major bleeding in patients with and without cancers.

AF, atrial fibrillation; BMI, body mass index; CI, confidence interval; COPD, chronic obstructive pulmonary disease; CKD, chronic kidney disease; HR, hazard ratio; VKA, vitamin K antagonist.


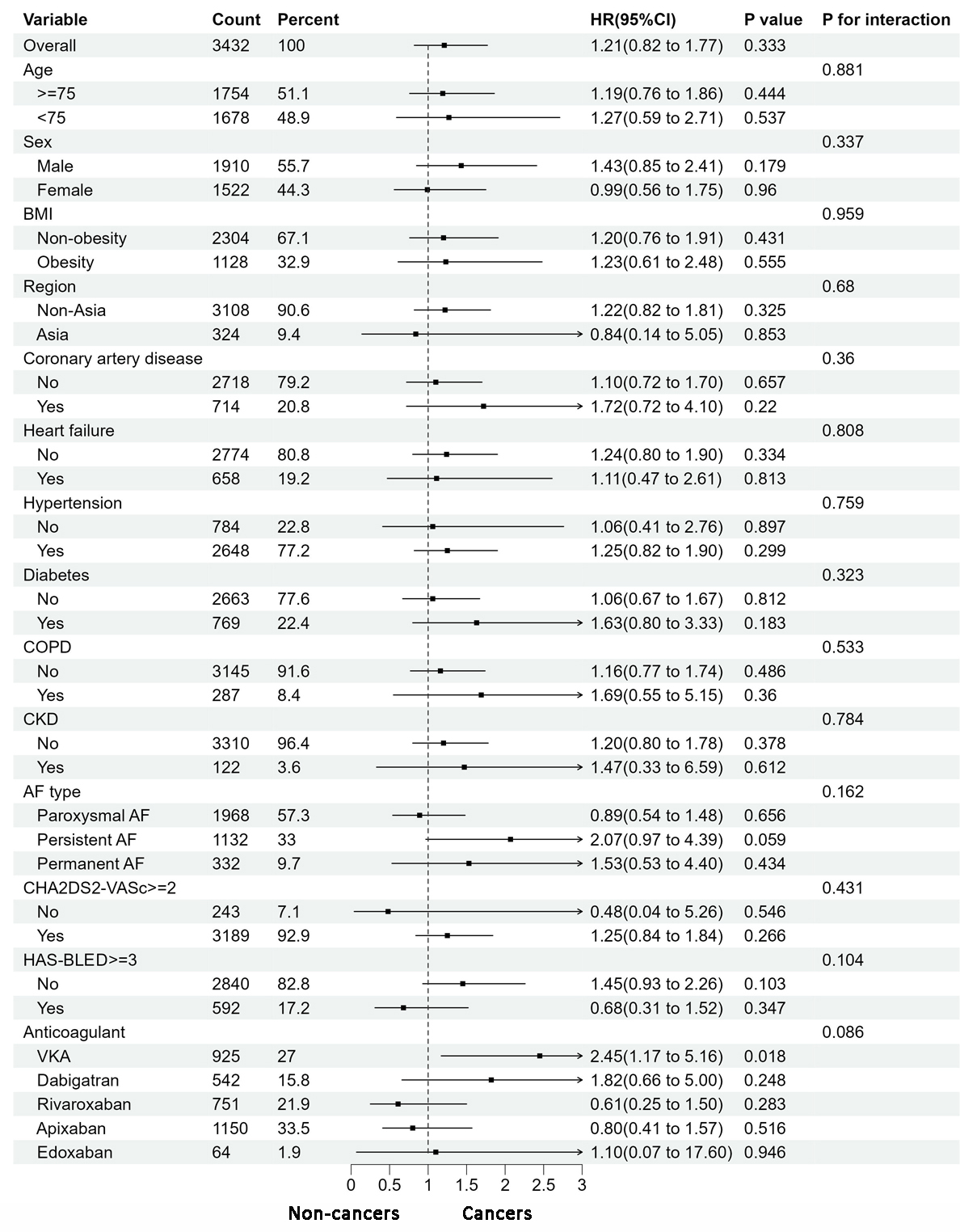


Supplement Figure 3: Subgroup analysis of thromboembolism in patients with and without cancers.

AF, atrial fibrillation; BMI, body mass index; CI, confidence interval; COPD, chronic obstructive pulmonary disease; CKD, chronic kidney disease; HR, hazard ratio; VKA, vitamin K antagonist.


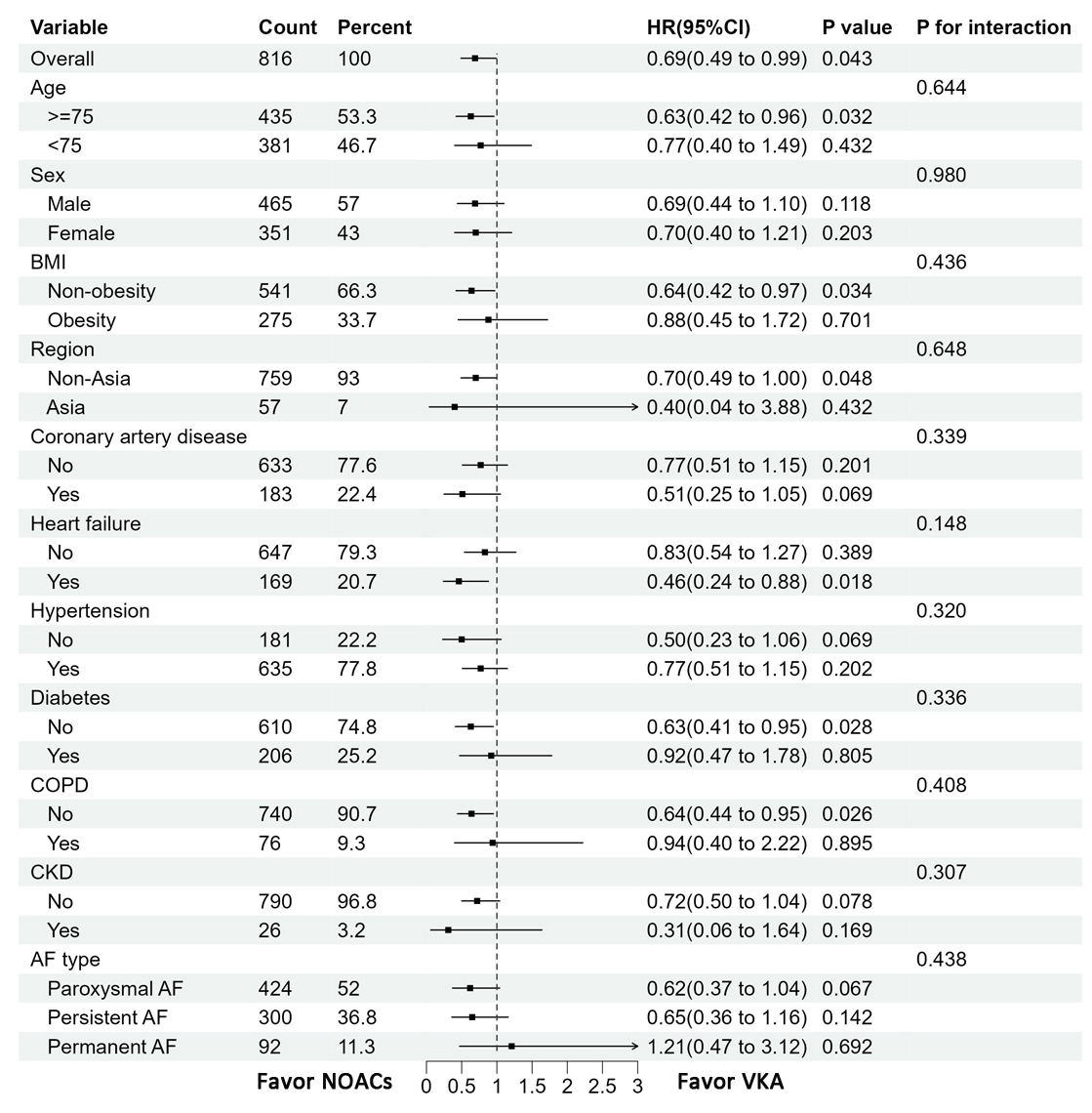


Supplement Figure 4: Subgroup analysis of the primary endpoint in patients treated with VKA and NOACs.

AF, atrial fibrillation; BMI, body mass index; CI, confidence interval; COPD, chronic obstructive pulmonary disease; CKD, chronic kidney disease; HR, hazard ratio.


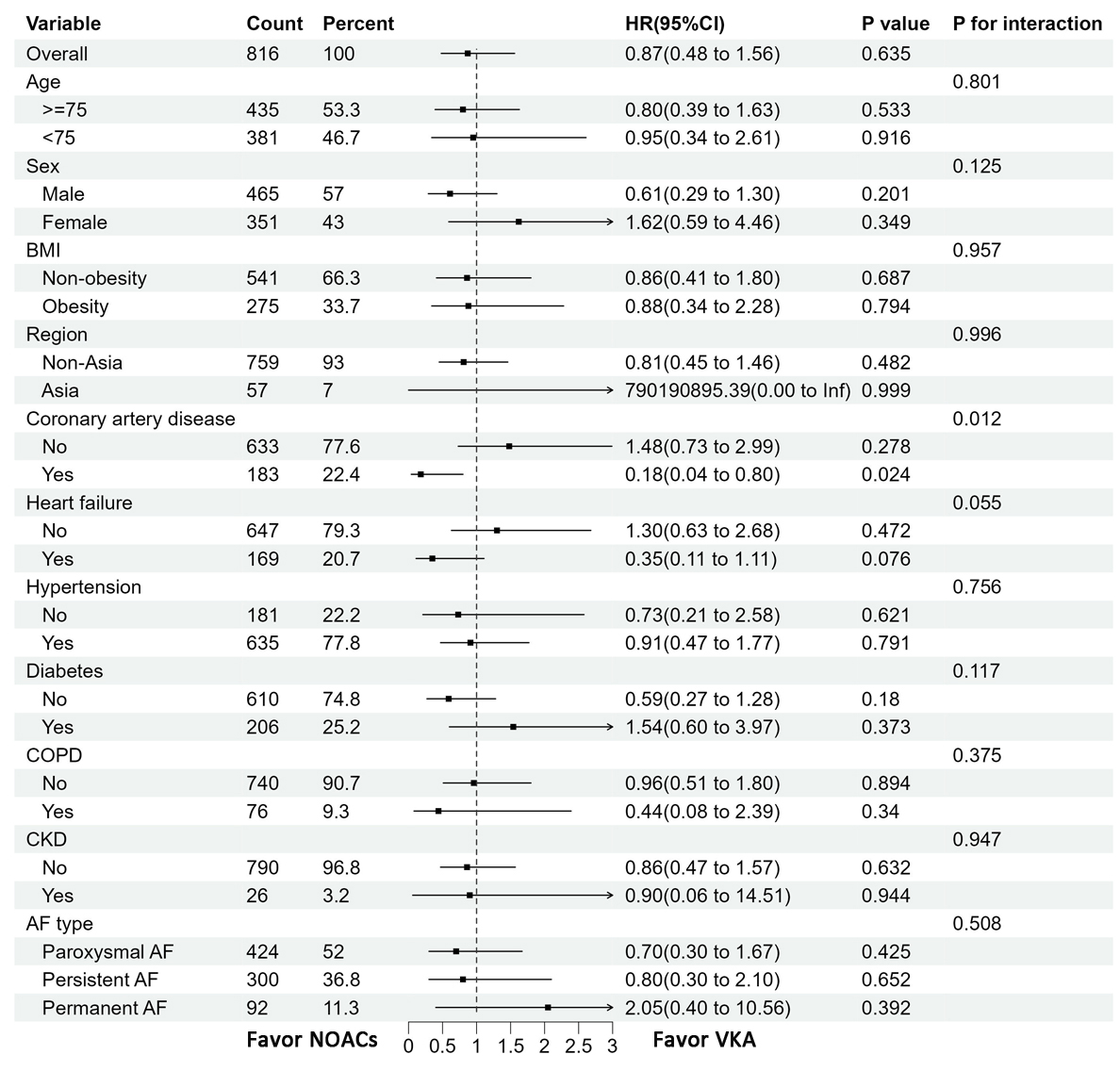


Supplement Figure 5: Subgroup analysis of major bleeding in patients treated with VKA and NOACs.

AF, atrial fibrillation; BMI, body mass index; CI, confidence interval; COPD, chronic obstructive pulmonary disease; CKD, chronic kidney disease; HR, hazard ratio.


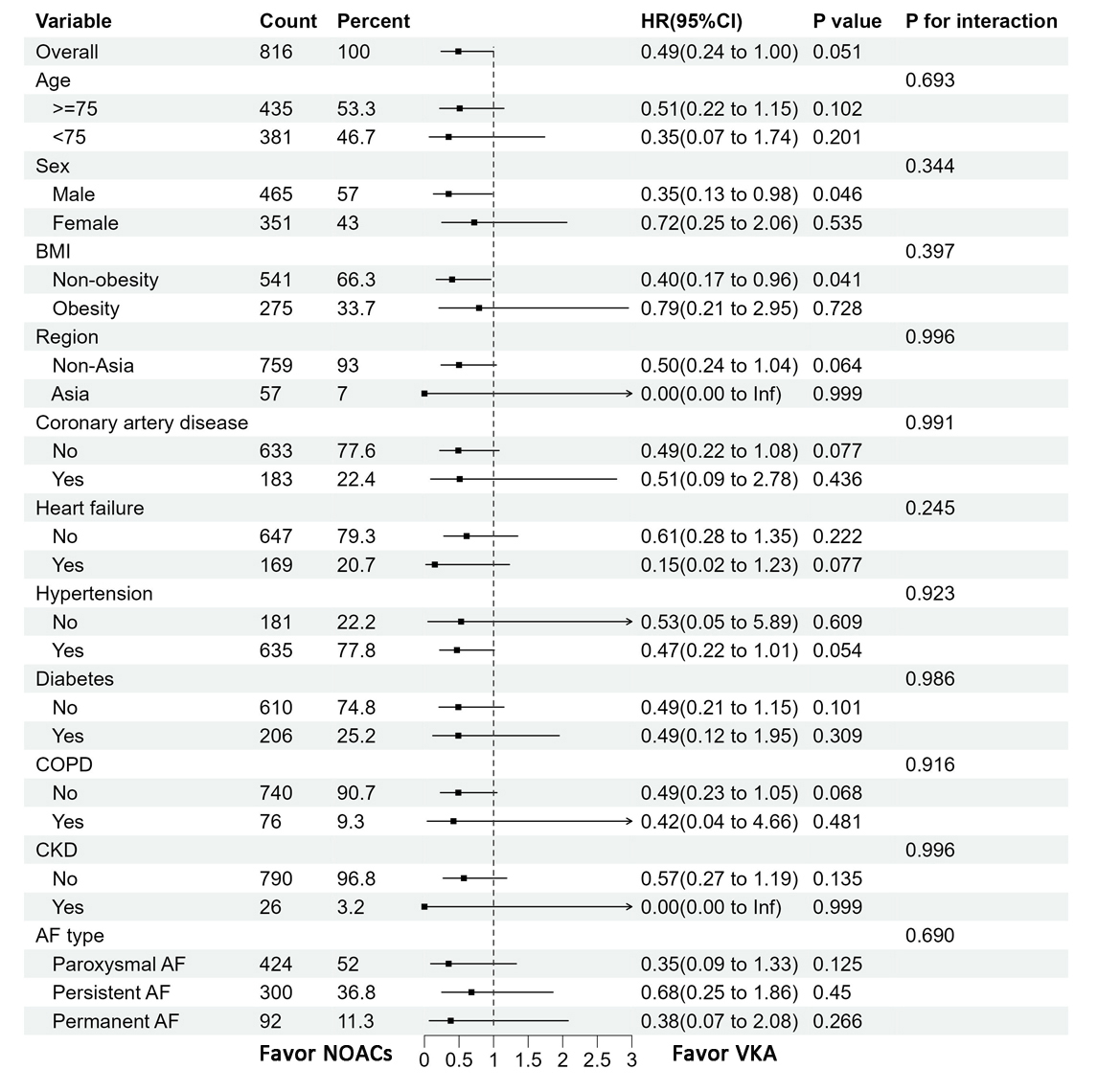


Supplement Figure 6: Subgroup analysis of thromboembolism in patients treated with VKA and NOACs.

AF, atrial fibrillation; BMI, body mass index; CI, confidence interval; COPD, chronic obstructive pulmonary disease; CKD, chronic kidney disease; HR, hazard ratio.
