# Supplementary material for: Long‐term risks and benefits of oral anticoagulation in atrial fibrillation patients with cancer: A report from the GLORIA‐AF registry
Source: Eur J Clin Invest. 2024 Nov 13;55(2):e14347. doi: 10.1111/eci.14347 (PMC11744914; doi:10.1111/eci.14347)
Supplement: Supplementary file 2 — Table S1: [file ECI-55-e14347-s001.docx]

Supplement Table 1. The related studies from GLORIA-AF Registry

| Reference | Topic | Conclusion |
| --- | --- | --- |
| Bergler-Klein J, et al^1^ | A descriptive analysis of antithrombotic usage, including three-year outcomes with Dabigatran and VKA for AF in Eastern Europe | Dabigatran was associated with reduced major bleeding, all-cause death, and cardiovascular composite, with comparable risk of stroke versus VKA |
| Lu J, et al^2^ | Predicting multifaceted risks using machine learning in AF | The multi-label gradient boosting decision tree model outperformed clinical risk scores in predicting the risks in patients with AF |
| Lam SHM, et al^3^ | The effect of digoxin and beta-blockers on cardiovascular outcomes and mortality among patients with atrial fibrillation | Combination therapy of beta-blockers and digoxin was associated with higher risks of MACE and all-cause death compared to beta-blockers alone |
| Romiti GF, et al^4^ | Phenotypes of AF patients according to comorbidities and impact on management and long-term prognosis | AF patients with complex phenotypes may require comprehensive and holistic approaches to improve their prognosis |
| Romiti GF, et al^5^ | Association between AF and COPD, in relation to treatment patterns and major outcomes | Patients with AF and COPD had worse outcomes, including higher mortality, MACE, and major bleeding |
| Romiti GF, et al^6^ | Association between clinical risk phenotypes on AF treatment patterns and the risk of major outcomes | In patients with AF, clinical risk phenotypes are multifaceted and heterogenous, and they are associated with differences in stroke prevention and worse prognosis |
| Corica B, et al^7^ | Association between metabolic status, BMI, and natural history of patients with AF | Prognosis was heterogeneous between BMI groups, with metabolically unhealthy patients showing higher risk of adverse events |
| Ding WY, et al^8^ | Validating the predictive ability of the 2MACE score for major adverse cardiovascular events in patients with AF | 2MACE score can adequately predict the risk of MACE in patients with AF |
| Romiti GF, et al^9^ | Impact of adherence to the ABC pathway on the risk of major adverse outcomes in a global cohort of patients with AF | Adherence to the ABC pathway in AF patients was associated with a reduced risk of major adverse events, including mortality, thromboembolism and MACE |
| Liu X, et al^10^ | Real world time trends in antithrombotic treatment for newly diagnosed atrial fibrillation in China | OAC prescriptions in Chinese patients with AF has increased over time, albeit with VKAs as the most common antithrombotic treatment. Most patients, including those at high stroke risk, remain undertreated |
| Beier L, et al^11^ | Evolution of antithrombotic therapy for patients with AF | More patients received NOACs in phase III compared to phase II. VKAs were preferred over NOACs in patients with impaired kidney function |
| Ding WY, et al^12^ | Incidence and risk factors for residual adverse events despite anticoagulation in AF | Patients with AF remain at significant residual risk of developing complications including ischemic stroke despite anticoagulation therapy |
| Ding WY, et al^13^ | Impact of early ablation of atrial fibrillation on long-term outcomes | Early AF ablation in AF patients who were predominantly treated with NOACs was associated with a survival advantage compared to medical therapy alone |
| Huisman MV, et al^14^ | Compare the safety and effectiveness of dabigatran versus VKA in patients with newly diagnosed AF | Dabigatran was associated with a 39% reduced risk of major bleeding and 22% reduced risk for all-cause death compared with VKA |
| Bayer V, et al^15^ | OAC use by geographical region and type of site in patients with recent-onset AF | Geographic variability exists with the use of OACs for patients with AF. There are differences in the time-to-treatment initiation of OAC by type of site |
| Kozieł M, et al^16^ | Changes in anticoagulant prescription patterns over time for patients with AF around the world | During 4 years after the approval of the first NOAC, NOAC use increased, while VKA use decreased, across all regions |
| Azar RR, et al^17^ | Antithrombotic treatment pattern in newly diagnosed AF patients and 2-year follow-up results for dabigatran-treated patients in the Africa/Middle-East Region | dabigatran was the most commonly used antithrombotic agent, which was associated with a high persistence rate and low incidence rates of stroke, MI, major bleeding, and all-cause mortality after 2 years of follow-up |
| Ishiguchi H, et al^18^ | Residual risks of thrombotic complications in anticoagulated patients with AF | Using hierarchical cluster analysis are associated with distinct residual thromboembolic risks and related outcomes |
| Liu Y, et al^19^ | Associations between adverse events and DM, as well as adverse events and sole insulin use | DM or individuals receiving insulin alone was independently associated with higher risks of all-cause death, cardiovascular death, MI, major bleeding and MACE in AF individuals |

GLORIA-AF, Global Registry on Long-Term Antithrombotic Treatment in Patients with Atrial Fibrillation; AF, atrial fibrillation; VKA, Vitamin K Antagonists; MACE, major cardiovascular events; COPD, chronic obstructive pulmonary disease; BMI, body mass index; ABC, Atrial Fibrillation Better Care; OAC, oral anticoagulant; NOACs, non-Vitamin K antagonist oral anticoagulants; MI, myocardial infarction; DM, diabetes mellitus.

**References**

1. Bergler-Klein J, Gotcheva N, Kalējs O, et al. Antithrombotic Usage, Including Three-Year Outcomes With Dabigatran and Vitamin K Antagonists for Atrial Fibrillation, in Eastern Europe: A Descriptive Analysis From Phase 3 of the GLORIA-AF Registry. *Am J Ther.* 2024;31(1):e1-e12.

2. Lu J, Bisson A, Bennamoun M, et al. Predicting multifaceted risks using machine learning in atrial fibrillation: insights from GLORIA-AF study. *Eur Heart J Digit Health.* 2024;5(3):235-246.

3. Lam SHM, Romiti GF, Olshansky B, Chao TF, Huisman MV, Lip GYH. Combination therapy of beta-blockers and digoxin is associated with increased risk of major adverse cardiovascular events and all-cause mortality in patients with atrial fibrillation: a report from the GLORIA-AF registry. *Intern Emerg Med.* 2024;19(5):1369-1378.

4. Romiti GF, Corica B, Mei DA, et al. Patterns of comorbidities in patients with atrial fibrillation and impact on management and long-term prognosis: an analysis from the Prospective Global GLORIA-AF Registry. *BMC Med.* 2024;22(1):151.

5. Romiti GF, Corica B, Mei DA, et al. Impact of chronic obstructive pulmonary disease in patients with atrial fibrillation: an analysis from the GLORIA-AF registry. *Europace.* 2023;26(1).

6. Romiti GF, Proietti M, Corica B, et al. Implications of Clinical Risk Phenotypes on the Management and Natural History of Atrial Fibrillation: A Report From the GLORIA-AF. *J Am Heart Assoc.* 2023;12(20):e030565.

7. Corica B, Romiti GF, Proietti M, et al. Clinical Outcomes in Metabolically Healthy and Unhealthy Obese and Overweight Patients With Atrial Fibrillation: Findings From the GLORIA-AF Registry. *Mayo Clin Proc.* 2024;99(6):927-939.

8. Ding WY, Fawzy AM, Romiti GF, et al. Validating the predictive ability of the 2MACE score for major adverse cardiovascular events in patients with atrial fibrillation: results from phase II/III of the GLORIA-AF registry. *J Thromb Thrombolysis.* 2024;57(1):39-49.

9. Romiti GF, Proietti M, Bonini N, et al. Adherence to the Atrial Fibrillation Better Care (ABC) pathway and the risk of major outcomes in patients with atrial fibrillation: A post-hoc analysis from the prospective GLORIA-AF Registry. *EClinicalMedicine.* 2023;55:101757.

10. Liu X, Feng G, Marler SV, Huisman MV, Lip GYH, Ma C. Real world time trends in antithrombotic treatment for newly diagnosed atrial fibrillation in China: reports from the GLORIA-AF Phase III registry : Trends in antithrombotic therapy use in China. *Thromb J.* 2023;21(1):83.

11. Beier L, Lu S, França LR, et al. Evolution of antithrombotic therapy for patients with atrial fibrillation: The prospective global GLORIA-AF registry program. *PLoS One.* 2022;17(10):e0274237.

12. Ding WY, Lane DA, Gupta D, Huisman MV, Lip GYH. Incidence and Risk Factors for Residual Adverse Events Despite Anticoagulation in Atrial Fibrillation: Results From Phase II/III of the GLORIA-AF Registry. *J Am Heart Assoc.* 2022;11(15):e026410.

13. Ding WY, Calvert P, Gupta D, Huisman MV, Lip GYH. Impact of early ablation of atrial fibrillation on long-term outcomes: results from phase II/III of the GLORIA-AF registry. *Clin Res Cardiol.* 2022;111(9):1057-1068.

14. Huisman MV, Teutsch C, Lu S, et al. Dabigatran versus vitamin K antagonists for atrial fibrillation in clinical practice: final outcomes from Phase III of the GLORIA-AF registry. *Clin Res Cardiol.* 2022;111(5):548-559.

15. Bayer V, Kotalczyk A, Kea B, et al. Global Oral Anticoagulation Use Varies by Region in Patients With Recent Diagnosis of Atrial Fibrillation: The GLORIA-AF Phase III Registry. *J Am Heart Assoc.* 2022;11(6):e023907.

16. Kozieł M, Teutsch C, Bayer V, et al. Changes in anticoagulant prescription patterns over time for patients with atrial fibrillation around the world. *J Arrhythm.* 2021;37(4):990-1006.

17. Azar RR, Ragy HI, Kozan O, et al. Antithrombotic treatment pattern in newly diagnosed atrial fibrillation patients and 2-year follow-up results for dabigatran-treated patients in the Africa/Middle-East Region: Phase II results from the GLORIA-AF registry program. *Int J Cardiol Heart Vasc.* 2021;34:100763.

18. Ishiguchi H, Abdul-Rahim AH, Huang B, et al. Residual Risks of Thrombotic Complications in Anticoagulated Patients with Atrial Fibrillation: A Cluster Analysis Approach from the GLORIA-AF Registry. *J Gen Intern Med.* 2024.

19. Liu Y, Chen Y, Lam SHM, et al. Diabetes mellitus and adverse clinical events in patients with atrial fibrillation: A report from the GLORIA-AF registry phase III. *Diabetes Obes Metab.* 2024.
